# Expression profiles and functions of ferroptosis-related genes in intimal hyperplasia induced by carotid artery ligation in mice

**DOI:** 10.3389/fgene.2022.964458

**Published:** 2022-08-30

**Authors:** Lina Zhang, Wei Li, Bo Shi, Xiaoqing Zhang, Kaizheng Gong

**Affiliations:** ^1^ Department of Cardiology, The Affiliated Hospital of Yangzhou University, Yangzhou University, Yangzhou, China; ^2^ School of Life Science, Liaoning Normal University, Dalian, China

**Keywords:** intimal hyperplasia, ferroptosis, in-stent restenois, atheroclerosis, biological analyses

## Abstract

Intimal hyperplasia (IH) is a prominent pathological event that occurs during in-stent restenosis and atherosclerosis. Ferroptosis, characterized by iron-dependent and lipid peroxidation, has become the recent focus of studies on the occurrence and progress of cardiovascular diseases. However, there are few studies on ferroptosis and IH. Therefore, we aimed to identify and validate ferroptosis-related markers in IH to explore new possibilities for IH diagnosis and treatment. The IH microarray dataset (GSE182291) was downloaded from the Gene Expression Omnibus (GEO) database and ferroptosis-related genes (FRGs) were obtained from the FerrDb databases. The differentially expressed genes (DEGs) were analyzed using the GEO2R. Overlapping was performed to identify the ferroptosis-related DEGs among the DEGs and FRGs. Then, clustering, Gene Ontology (GO), Kyoto Encyclopedia of Genes and Genomes (KEGG) pathway enrichment, and protein–protein interaction (PPI) analyses were performed. Subsequently, the hub genes were identified using Cytoscape and hub gene–transcription factors and hub gene–microRNA networks were constructed. Finally, real-time qPCR (RT-qPCR) and immunohistochemistry (IHC) were used to verify the mRNA and protein levels of the hub FRGs in IH. Thirty-four FRGs showing significantly different expression were identified from a total of 1,197 DEGs 2 days after ligation; 31 FRGs were selected from a total of 1,556 DEGs 14 days after ligation. The GO and KEGG analyses revealed that these 34 ferroptosis-related DEGs identified 2 days after ligation were mainly enriched in the basolateral plasma membrane, ferroptosis, lipid and atherosclerosis, and IL-17 signaling pathways. The 31 ferroptosis-related DEGs in endometrial hyperplasia identified 14 days after ligation were mainly enriched in response to oxidative stress, ferroptosis, tumor necrosis factor signaling pathway, and lipid and atherosclerosis. Five hub FRGs (Il1b, Ptgs2, Cybb, Cd44, and Tfrc) were identified using PPI networks; four hub FRGs (Il1b, Ptgs2, Cybb, and Cd44) were validated to be upregulated 2 and 14 days after ligation using RT-qPCR and show significantly different expression 14 days after ligation via IHC. Our findings verify the expression of hub DEGs related to ferroptosis in IH and elucidate the potential relationship between ferroptosis and IH, providing more evidence about the vital role of ferroptosis in IH.

## 1 Introduction

Intimal hyperplasia (IH) is a principal pathophysiological process of early atherosclerosis, in-stent restenosis, and vein bypass graft failure (Dzau and Braun-Dullaeus 2002). It is triggered by endothelial damage and leads to progressively increasing luminal narrowing or restenosis, which sometimes proves fatal. Previous studies have shown that the occurrence and development of IH is regulated by various biological mechanisms, such as autophagy, epigenetics, oxidative stress, and endoplasmic reticulum stress (Xue and Chen, 2019). To date, studies on IH have focused primarily on the vascular smooth muscle cell (VSMC) proliferation pathway of IH development. Limitations, such as unclear pathogenesis and few biological datasets, have spurred the search for new target genes and efficient approaches to control IH.

Ferroptosis is a new type of cell death that is distinct from apoptosis, autophagy, and necrosis in morphology and function, and is characterized by iron overload and lipid peroxidation (Dixon and Lemberg 2012). Previous studies have shown that ferroptosis is involved in many diseases, including tumors and neurodegenerative disorders (e.g., Alzheimer’s disease and Parkinson’s disease) (Do and Gouel, 2016; Hao and Yu, 2017; Lane and Ayton, 2018). Recent studies have also shown that ferroptosis is involved in most cardiovascular diseases (CVDs), such as cardiomyopathy (Fang and Wang, 2019), myocardial infarction (Baba and Higa, 2018), ischemia/reperfusion injury (Stamenkovic and O'Hara, 2021), heart failure (Fang and Cai, 2020), and atherosclerosis (Bai and Li, 2020). Several studies have found that targeting ferroptosis can serve as a feasible approach for preventing cardiomyocyte death and managing cardiac pathologies (Ravingerova and Kindernay, 2020; Wu and Li, 2021).

However, to the best of our knowledge, there are only few studies on the function of ferroptosis in the pathological process of IH, which highlights the novelty of our study. Therefore, in the present study, we aimed to identify and validate ferroptosis-related markers in IH to explore new possibilities for IH diagnosis and treatment.

## 2 Materials and methods

### 2.1 Microarray data

The microarray expression dataset (GSE182291) was downloaded from the Gene Expression Omnibus (GEO) database (https://www.ncbi.nlm.nih.gov/geo/). Five groups of tissue samples from the right and left carotid arteries were analyzed 2 and 14 days after ligation, respectively. The data were based on the GPL11180 platforms (Affymetrix HT MG-430 p.m. Array Plate).

Identification of differentially expressed genes (DEGs) related to ferroptosis

DEGs were identified using the GEO2R online analysis tool. The classical Bayesian test in the limma package was used to perform differential expression analysis on the two groups of samples. Genes with a |log2FC| ≥1 (FC: fold change) and adjusted *p*-value of <0.05 were defined as DEGs. Ferroptosis-related gene (FRG) sets were acquired from FerrDb (http://www.zhounan.org/ferrdb/index.html). The overlap was performed to differentiate the ferroptosis-related DEGs from the DEGs and FRGs.

Gene ontology (GO) terms and pathway enrichment analysis for ferroptosis-related DEGs

Based on DAVID v.6.8 (the Database for Annotation, Visualization, Integrated Discovery), GO (including biological processes, cellular components, and molecular functions) and Kyoto Encyclopedia of Genes and Genomes (KEGG) pathway analyses were used to analyze the functions and related pathways of ferroptosis-related DEGs. A *p*-value < 0.05 was set as statistically significant.

### 2.2 Protein–protein interaction (PPI) establishment and identification of hub genes

The PPI networks of ferroptosis-related DEGs 2 and 14 days after ligation were assessed using the PPI network analysis on the STRING online tool (https://cn.string-db.org/) and visualized using Cytoscape.

### 2.3 Construction of microRNA (miRNA)-mRNA and TF-mRNA networks

MiRNAs and transcription factors (TFs) exert their biological functions by regulating the expression of target mRNA. Hence, we used the intersection between three databases, namely miRWALK (http://mirwalk.umm.uni-heidelberg.de), miRDB (http://www.mirdb.org/), and Targetscan (http://www.targetscan.org), to predict the potential target miRNAs of hub FRGs. The TF of hub FRGs were predicted using the TRRUST2.0 online tool (https://www.grnpedia.org/trrust/). In addition, miRNA-mRNA and TF-mRNA networks were visualized using Cytoscape. The hub genes in the miRNA-mRNA network were obtained using the cytoHubba plugin.

### 2.4 Carotid artery ligation model

Male, specific pathogen free C57BL/6 mice (8–12 weeks, 20–25 g) were purchased from the Yangzhou University (China). To exclude the effects of estrogen on vascular damage, only adult male mice were used in the study following a 7-days acclimatization period to the preoperative environment. Briefly, the samples were divided into two groups, the ligation group included the left carotid arteries (LCAs) whereas the intra animal control group included the contralateral right carotid arteries (RCAs) on which a sham operation was performed. For carotid artery ligation, ketamine (80 mg/kg intraperitoneal) and xylazine (5 mg/kg intraperitoneal) were combined to anesthetize mice and the LCA was exposed through a midline cervical incision and ligated with a 5–0 silk suture just proximal to the bifurcation. A similar procedure was performed but without ligation on the RCA. Total vascular tissue samples were obtained from the LCAs and RCAs of mice sacrificed at 2 and 14 days post-ligation. Then, the mice were processed for morphological and biochemical studies at specific time points after surgery, as described previously (Zhang and Gu, 2022). All protocols in this study were approved by the Institutional Animal Care and Use Committee of the Affiliated Hospital of Yangzhou University, and followed the Guide for the Care and Use of Laboratory Animals.

### 2.5 Real-time qPCR(RT-qPCR)

The mRNA expression levels of the FRGs were measured using RT-qPCR; the primers were designed by the NCBI website and synthesized through the Tsingke Biotechnology Company of China. At 2 and 14 days after ligation, total RNA from vascular tissues was extracted using the TRIzol universal Reagent (Tiangen), which was then reverse-transcribed into cDNA using HiScript^®^ Ⅲ RT SuperMix for qPCR (+gDNA wiper) (NOVIZAN). Synthesized cDNA was amplified through quantitative RT-PCR analysis using ChamQ universal SYBR qPCR Master Mix (NOVIZAN) in a CFX96 Real-Time System (Bio-Rad). Accordingly, the relative abundance of each transcript was determined using the ΔΔCT method. The forward and reverse primer pairs used for quantitative RT-qPCR are shown in Table 1.

**TABLE 1:** Forward and reverse primer pairs.

Gene names	Forward	Reverse
Mus-Tfrc	CTTCGCAGGCCAGTGCT	TAC​AAG​GGA​GTA​CCC​CGA​CA
Mus-Ptgs2	CAT​CCC​CTT​CCT​GCG​AAG​TT	GGC​CCT​GGT​GTA​GTA​GGA​GA
Mus-Cybb	CCC​TCC​CTG​TCT​AGG​TAA​TGC	GCA​TTT​GCC​TTC​GGT​GAT​GT
Mus-IL-1b	CCA​CCT​CAA​TGG​ACA​GAA​TAT​CA	CCC​AAG​GCC​ACA​GGT​ATT​T
Mus-Cd44	GCA​GAA​ATC​AAG​ACG​TTA​TGG​G	AAGCACCACCACCAAAGA

### 2.6 Immunohistochemistry (IHC)

IHC was performed as previously described (Zhang and Gu, 2022). Briefly, 5-µm thick formalin-fixed paraffin-embedded carotid tissue of mice sections were stained with anti-Cd44 (Servicebio), anti-Il1b (Servicebio), anti-Ptgs2 (Servicebio), and anti-Cybb (Servicebio) antibodies according to the manufacturer’s instructions. All positive cells were counted from three sections of each artery sample and evaluated by an investigator who was blinded to the identities of the treatment protocols at ×100 magnification.

### 2.7 Statistical analysis

The data were expressed as the mean ± SD in GraphPad Prism 7 (GraphPad Software). A two-sample, unpaired Student’s t-test was used to analyze the differences between the two groups of data with normally distributed variables and the probability level was set at *p* < 0.05.

## 3 Results

**FIGURE 1:**
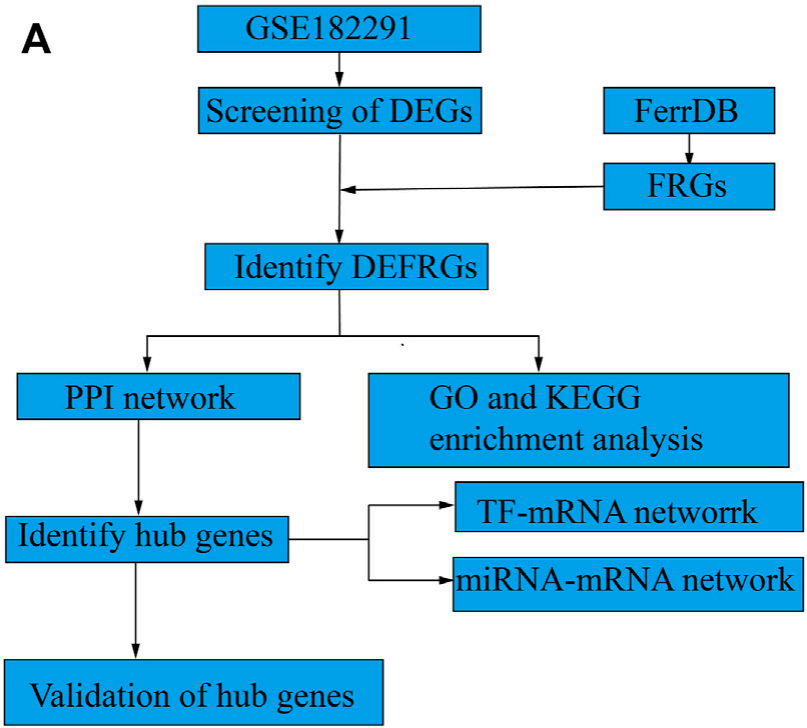
Study protocol. **(A)** The overall protocol of this study.

To perform an in-depth analysis of ferroptosis-associated genes in IH, gene expression and FRG datasets from GEO and FerrDb were used, respectively. An overview of the datasets analyzed and compared in this study is shown in Figure 1 (study protocol).

### 3.1 Ferroptosis-related DEGs in IH

**FIGURE 2:**
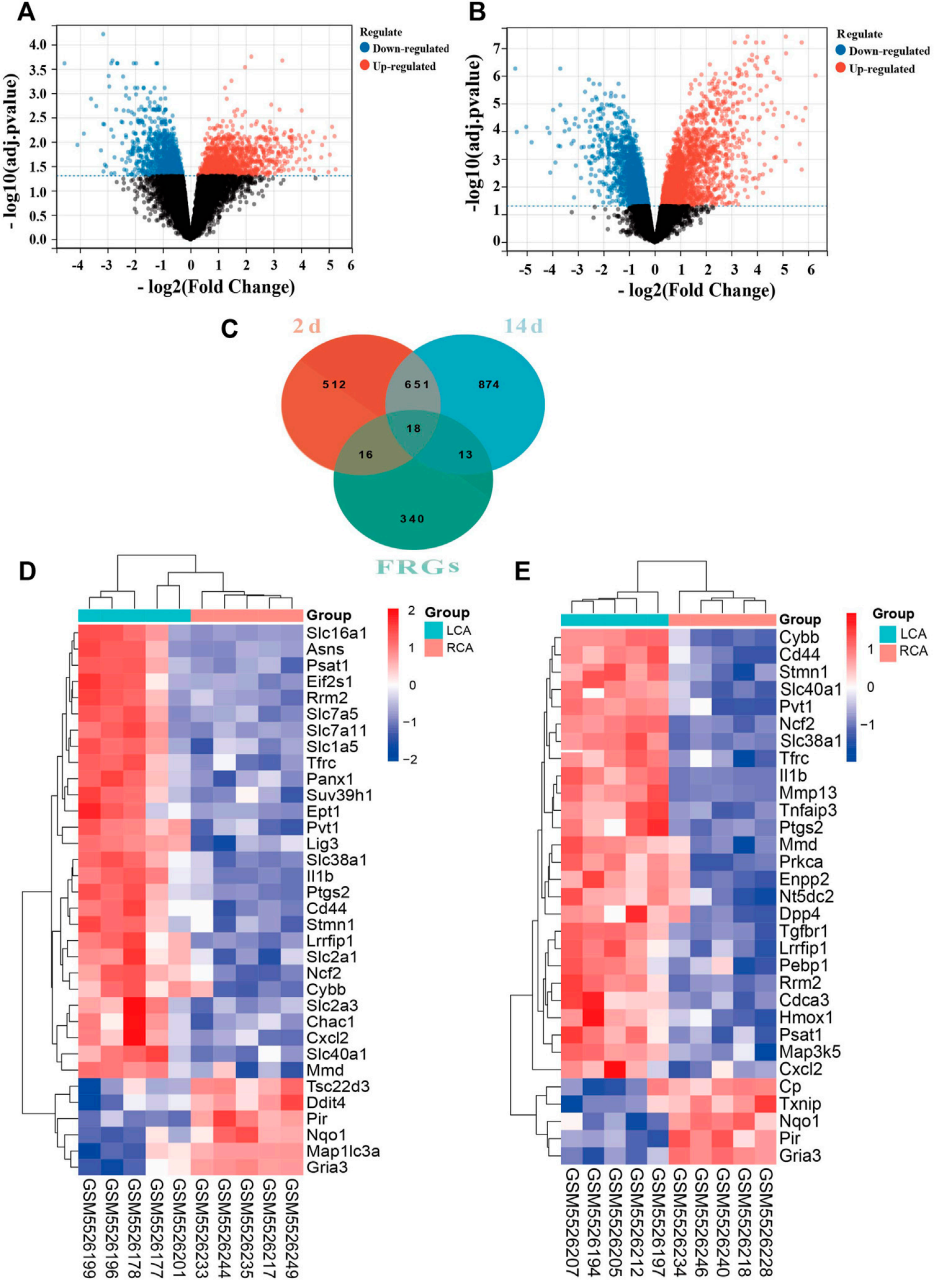
Differentially expressed ferroptosis-related genes (FRGs) in early and late intimal hyperplasia (IH). **(A)** The volcano plot of differentially expressed genes 2 days after ligation. **(B)** The volcano plot of differentially expressed genes 14 days after ligation. The abscissa represents the difference in the fold change of gene expression in different treatment groups, and the ordinate represents the adj. *p*-value of the expression difference. Blank dots represent unchanged genes. Red dots represent upregulated genes, and blue dots represent downregulated genes. **(C)**The overlapping genes between FRGs and DEGs 2 and 14 days after ligation. **(D)** The heatmap of differentially expressed FRGs in the carotid artery samples 2 days after ligation based on the clustering analysis. **(E)** The heatmap of differentially expressed FRGs in the carotid artery samples 14 days after ligation based on the clustering analysis.

We found that the expression of a larger number of genes were altered in the endothelium of the LCA than the RCA. A total of 1,197 and 1,556 DEGs were identified 2 and 14 days after ligation, respectively, as shown in Figures 2A,B. There were 388 FRGs in FerrDb; however, after intersection with FRGs, 34 ferroptosis-related DEGs were identified 2 days after ligation and 31 ferroptosis-related DEGs were identified 14 days after ligation, as shown in Figure 2C. The clustering analysis of significantly different FRGs 2 and 14 days after ligation showed that the samples were closely related, as shown in Figures 2D,E.

### 3.2 Functional enrichment analysis of ferroptosis-related DEGs

**FIGURE 3:**
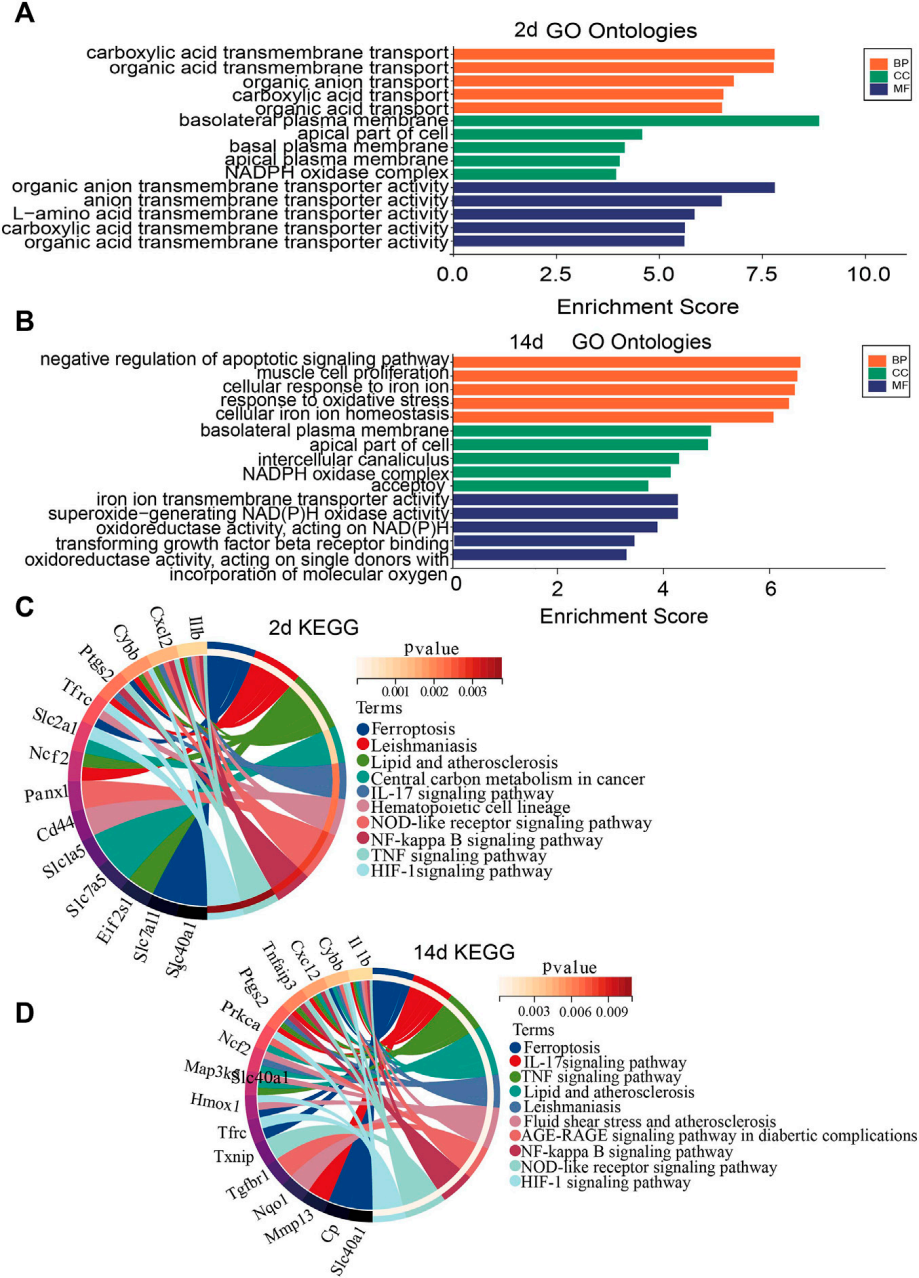
Gene Ontology (GO) and Kyoto Encyclopedia of Genes and Genomes (KEGG) enrichment analysis of FRGs. **(A)** GO enrichment analysis of FRGs 2 days after ligation. **(B)** GO enrichment analysis of FRGs 14 days after ligation. **(C)** Relationship among the top 10 enriched KEGG pathway terms and targets is represented in a chord plot 2 days after ligation. **(D)** Relationship among the top 10 enriched KEGG pathway terms and targets is represented in a chord plot 14 days after ligation. BP, biological processes; CC, cellular component. MF, molecular function.

To investigate the biological functions and pathways of ferroptosis-related DEGs 2 and 14 days after ligation, GO and KEGG enrichment analyses were performed, respectively. The GO analysis showed that differentially expressed FRGs were mainly enriched in basolateral plasma membrane, organic anion transmembrane transporter, and carboxylic acid transmembrane transport 2 days after ligation (Figure 3A). In addition, the KEGG results showed that the differentially expressed FRGs were closely enriched in ferroptosis, leishmaniasis, and lipid and atherosclerosis (Figure 3C). The GO analysis 14 days after ligation showed that differentially expressed FRGs were mainly enriched in the negative regulation of apoptotic signaling pathway, cellular response to iron ion, and response to oxidative stress (Figure 3B). Furthermore, the KEGG results showed that the differentially expressed FRGs were mainly enriched in ferroptosis, IL-17 signaling pathway, TNF-signaling pathway, and lipid and atherosclerosis (Figure 3D).

### 3.3 PPI networks and prediction of TFs for DEGs in mouse

**FIGURE 4:**
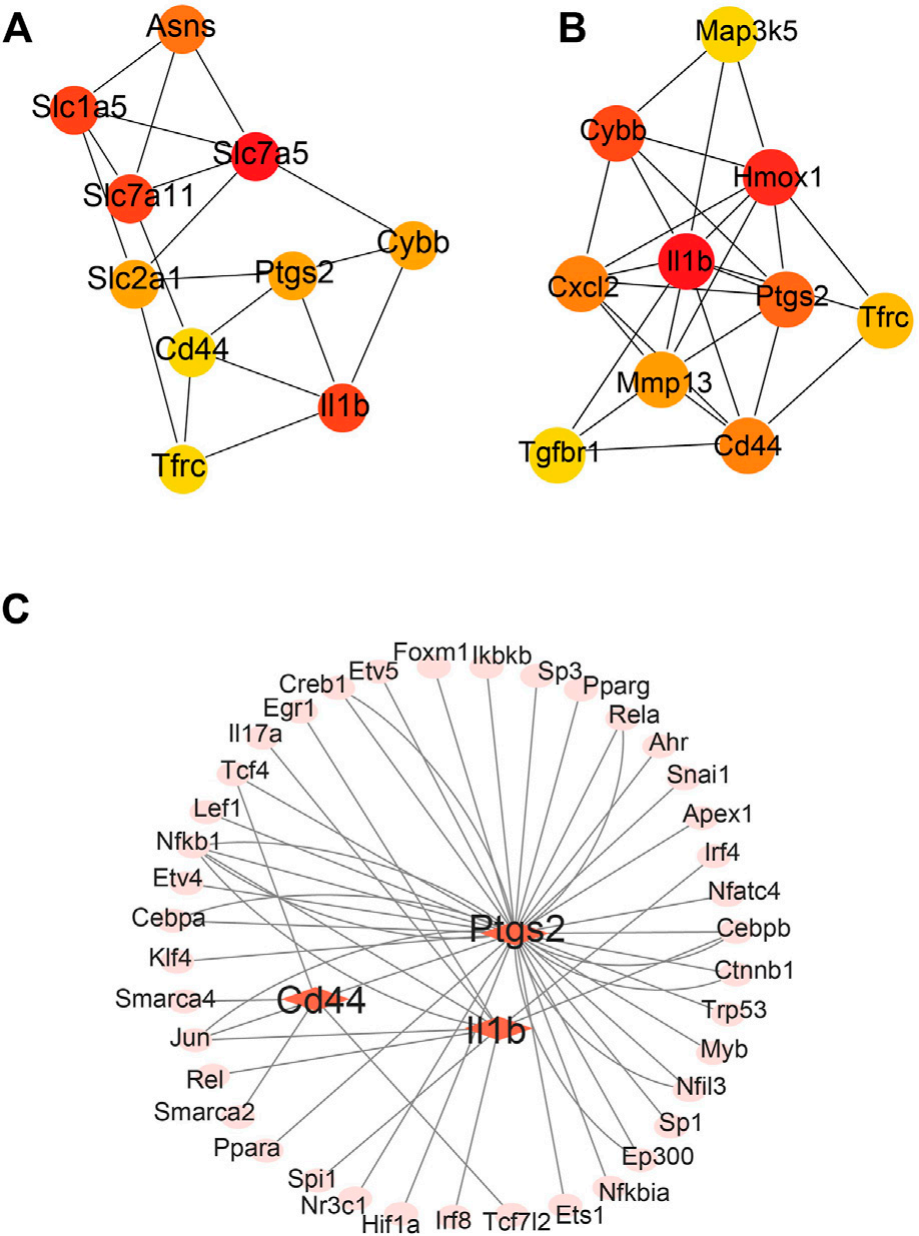
Major protein–protein interaction (PPI) networks and predicted transcription factors (TFs) of FRGs. **(A)** The major PPI network analysis of FRGs 2 days after ligation. **(B)** The major PPI network analysis of FRGs at 14 days after ligation. The color gradation represents the expression; red represents a higher expression. **(C)** The prediction of mouse DEGs TFs.

The differentially expressed FRGs 2 and 14 days after ligation were analyzed using the STRING online database and a PPI network was obtained (Supplementary Figure S1A, B). To identify hub FRGs, the CytoHubba plugin was used. The top 10 hub FRGs 2 days after ligation included Slc7a5, Il1b, Slc7a11, Slcla5, Asns, Ptgs2, Cybb, Slc2al, Cd44, and Tfrc (Figure 4A). However, 14 days after ligation, the top 10 hub FRGs included Il1b, Hmox1, Cybb, Ptgs2, Cd44, Cxcl2, Mmp13, Tfrc, Tgfbr1, and Map3k5 (Figure 4B). Moreover, we found that Il1b, Cybb, Tfrc, Cd44, and Ptgs2 co-existed and were upregulated in the LCA compared to the RCA at 2 and 14 days after ligation. To determine target-regulated FRG TFs, we used the TRRUST2.0 database and found that 4, 10, and 40 TFs regulate the expression of Cd44, Il1b, and Ptgs2, respectively, however, there were no TFs regulating Cybb and Tfrc (Figure 4C).

### 3.4 Construction of miRNA-mRNA networks

**FIGURE 5:**
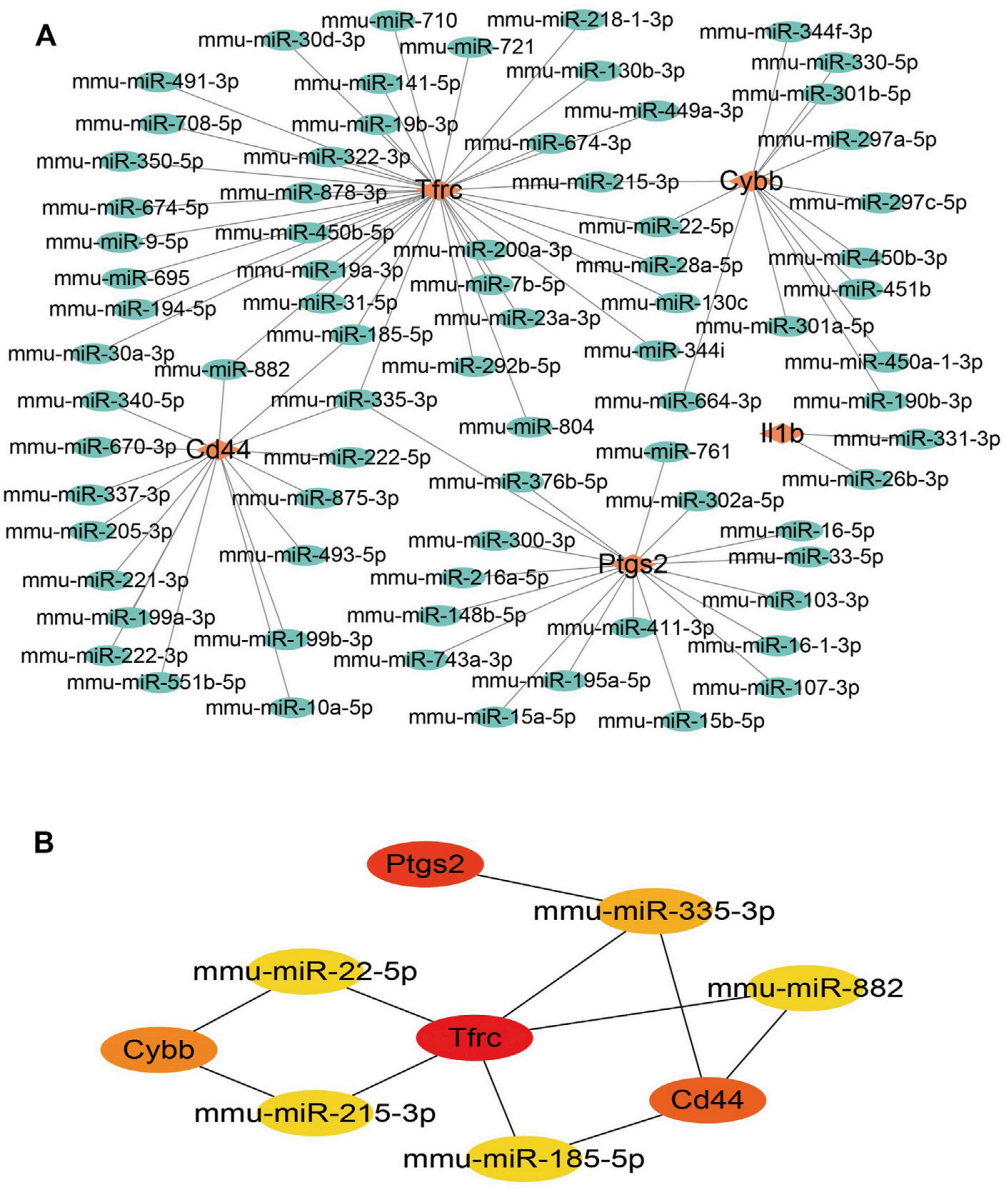
miRNA-mRNA network construction of hub genes. **(A)**miRNA-mRNA network construction. **(B)** The hub genes in the miRNA-mRNA network.

To ensure the accuracy and reliability of the results, the intersection of three databases (miRDB, Targetscan, and miRWALK) was selected to identify target-regulated hub gene miRNAs. By analyzing the miRNA–mRNA networks, we found that 8, 50, 79, 42, and 48 miRNA targets regulate the expression of Il1b, Cd44, Tfrc, Ptgs2, and Cybb, respectively (Figure 5A). Furthermore, miRNA-mRNA networks showed that miR-335-3p simultaneously regulates the expression of Ptgs2, Tfrc, and Cd44, miR-882 and miR-185-5p regulate the expression of Cd44 and Tfrc, miR-22-5p and miR-215-3p regulate the expression of Tfrc and Cybb (Figure 5B).

### 3.5 Hub gene validation

**FIGURE 6:**
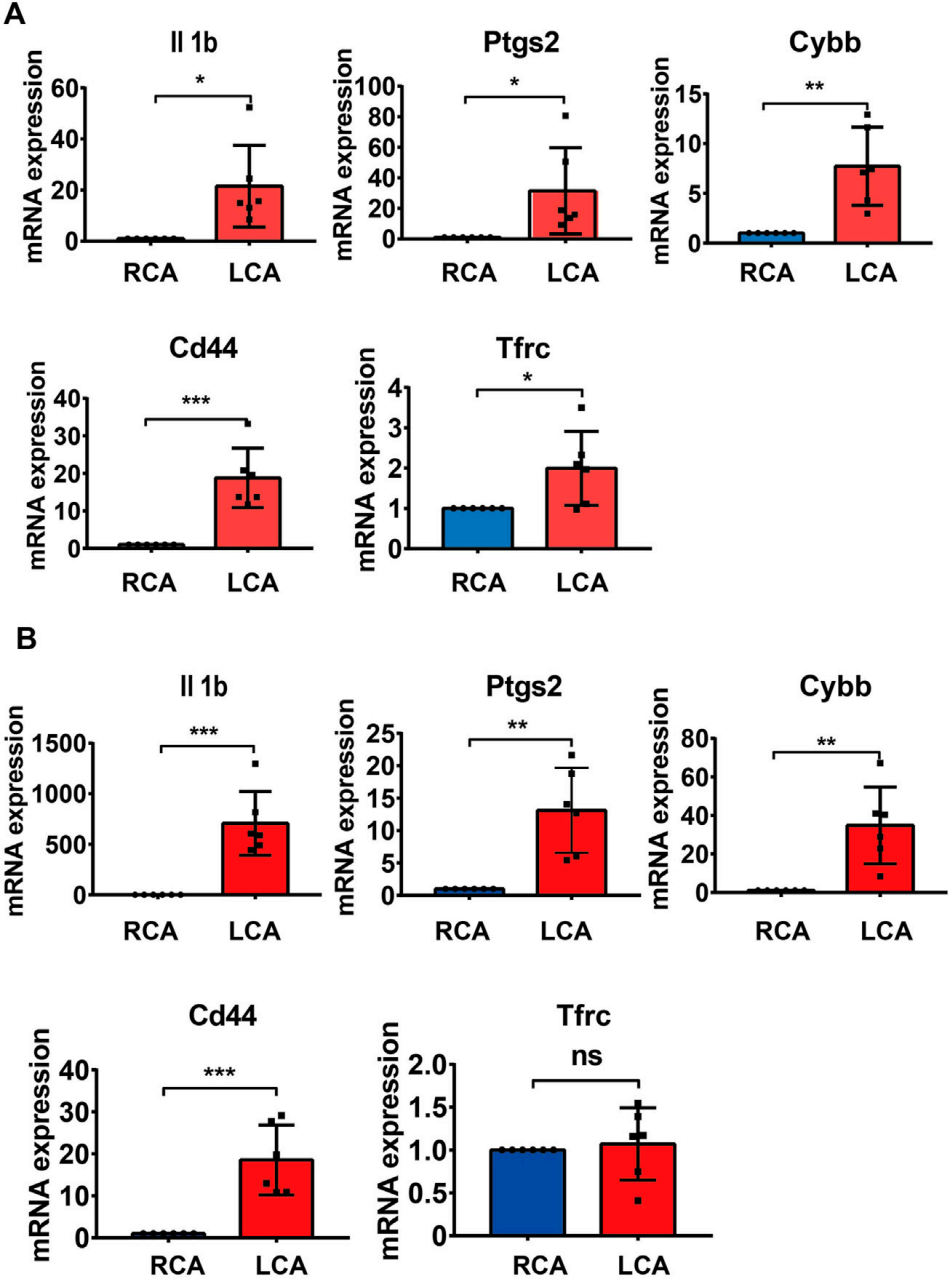
Validation of mRNA levels. **(A)** The mRNA levels of Il1b, Ptgs2, Cybb, and Tfrc 2 days after ligation in the right carotid artery (RCA) and left carotid artery (LCA) groups in mice. **(B)** The mRNA levels of Il1b, Ptgs2, Cybb, Cd44, and Tfrc 14 days after ligation in the RCA and LCA groups in mice. All values have been standardized using the expression levels of GAPDH. A two-tailed unpaired Student’s t-test was used to compare two groups. Data are expressed as the means ± SD, *n* = 6, *p < 0.05; **p < 0.01; ***p < 0.001; ****p < 0.0001.

**FIGURE 7:**
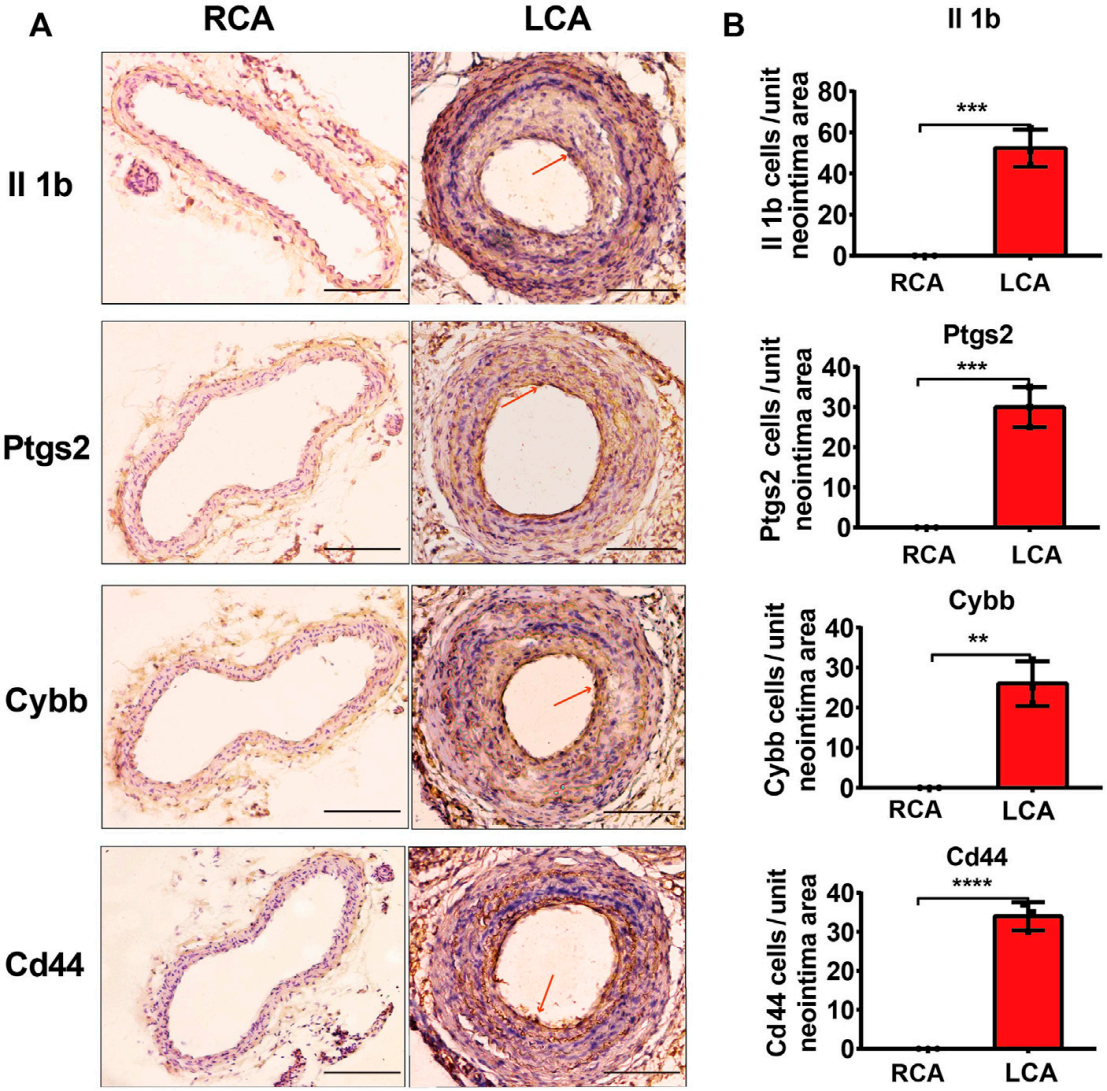
Validation of protein levels. **(A)** Immunohistochemistry staining of Il1b, Ptgs2, Cybb, and Cd44 proteins 14 days after ligation in the RCA and LCA groups in mice. **(B)** Quantitative analysis of Il1b, Ptgs2, Cybb, and Cd44 proteins 14 days after ligation in the RCA and LCA groups in mice. The arteries were harvested from uninjured RCA (that underwent a sham operation) and injured LCA 14 days after ligation. The red arrows represent positive cells. A two-tailed unpaired Student’s t-test was used to compare the two groups. Data are expressed as the means ± SD, *n* = 3. **p < 0.01; ***p < 0.001; ****p < 0.0001; compared with the RCA group. Original magnification, ×100. Scale bar: 50 μm.

To further validate the results of the hub DEGs related to ferroptosis analyzed from bioinformatics analysis, a model of IH was established by performing a carotid artery ligation in mice. Quantitative RT-PCR analysis showed that the mRNA levels of Il1b, Cybb, Ptgs2, Tfrc, and Cd44 were significantly higher (*p* < 0.05) in the LCA than in the RCA group 2 days after ligation. However, with the exception of Tfrc, the expression of Il1b, Cybb, Ptgs2, and Cd44 was significantly higher in the LCA than in the RCA group 14 days after ligation (Figure 6). Subsequently, we validated the presence of Il1b, Cybb, Ptgs2, and Cd44 using IHC (Figure 7) and found that the expression levels of these FRGs protein were elevated in LCA mice 14 days after ligation, compared with RCA mice. In addition, the quantification table of mRNA and IHC showed that the rising trend of Il-1b in LCA was more evident than that in RCA, which supported the findings of the bioinformatics analysis.

## 4 Discussion

Studies have shown that endothelial cell injury activation, monocyte/macrophage adhesion, and infiltration, are the main pathological bases of IH (Morrell and Adnot, 2009; Weber and Noels, 2011; Nazari-Jahantigh and Wei, 2012; Andueza and Kumar, 2020). When endothelial cells are injured, lipid metabolism is disordered, and lipids are gradually deposited in the intima. Oxidized lipids and lipid aggregation lead to the activation of macrophages, thus promoting the formation of foam tissue cells, which is the core process of atherosclerosis. Ferroptosis, an iron-dependent, non-apoptotic mode of cell death, is characterized by the accumulation of lipid reactive oxygen species (ROS) (Dixon and Lemberg, 2012). Recent studies revealed that ferroptosis plays a key role in the progression of atherosclerosis (Guo and Lu, 2022). Thus, elucidating the potential relationship between ferroptosis and IH may provide new ideas and targets for the in-depth study of IH. Kumar et al. mainly investigated the effects of atorvastatin at two time points on global endothelial gene expression by performing microarray studies using their mouse partial carotid ligation model (Kumar and Sur, 2021). In our study, there were four time points in the dataset; however, a comprehensive analysis found no intersection across these, and FRGs were very few at 12 and 24 h, which not enough to support the following functional enrichment and pathway analysis. Therefore, we systematically analyzed the expression of FRGs in the carotid artery samples 2 and 14 days post-ligation. It was found that 34 significantly different FRGs were identified 2 days after ligation, and 31 FRGs were identified 14 days after ligation. Then, GO enrichment analysis and KEGG separately revealed the diversity of functions and pathways of FRGs. Although, the specific role of these FRGs in IH requires further study, we speculated that these genes may play key roles in the pathophysiological processes of IH. Thus, carotid artery ligation altered the gene expression profile of endothelial cells, and although not many FRGs were obtained at days 2 and 14, their effects were mainly reflected in ferroptosis, immune inflammation, and lipid and atherosclerosis. Furthermore, to identify potential ferroptosis-related candidate target genes to enrich potential targets, we analyzed these genes through PPI network and identified five ferroptosis-related DEGs, including Il1b, Ptgs2, Cybb, Cd44, and Tfrc.

Inflammation is an important driver of atherosclerosis and the underlying pathology of CVDs. The NLRP3 inflammasome and IL-1 family of cytokines are central to the pathologic response to injury and represent a key pathogenetic mechanism in the formation, progression, and complication of atherosclerosis and the myocardial response to ischemic and non-ischemic injuries. IL-1-targeted therapies have been shown to improve cardiovascular outcomes in clinical trials in patients with or at risk for acute myocardial infarction, heart failure, and recurrent pericarditis (Ridker and Everett, 2017; Grebe and Hoss, 2018; Abbate and Toldo, 2020). Recent studies have revealed that NLRP3 inflammasome activation contributes to not only pyroptosis but also other types of cell death, including apoptosis, necroptosis, and ferroptosis (Huang and Xu, 2021). In addition, GPX4, as an important negative effector of ferroptosis, has recently been shown to inhibit caspase-11-dependent pyroptosis and IL-1β release (Kang and Zeng, 2018). Furthermore, a recent study showed that when ox-LDL and ferric ammonium citrate (FAC) were added to THP-1 macrophages, FAC, as an iron additive, increased the levels of lipid ROS, ferroptosis, IL-1β, and IL-18 in foam cells but decreased GPX4 expression (Su and Yang, 2021). These findings suggested that IL-1β may reflect the severity of ferroptosis. It has been previously reported that the expression of prostaglandin endoperoxide synthase 2 (Ptgs2) encoding cyclooxygenase-2 is significantly upregulated in ferroptosis (Xie and Hou, 2016). Previously, Li et al. investigated the role and underlying mechanisms of ferroptosis in lipopolysaccharide (LPS)-induced cardiac injury. They found that LPS increased levels of ferroptosis markers, including Ptgs2, malondialdehyde (MDA), and lipid ROS in mice injected with LPS (10 mg/kg) after 12 h (Li and Wang, 2020). Upregulation-trends of Ptgs2 in ferroptosis was further demonstrated in the study by Zhou et al. In this study, they found that the expression of PTGS2, ACSL4, caspase-1, and NLRP3 were upregulated at the late stages of atherosclerosis, and these proteins could be used as biomarkers of atherosclerosis severity (Zhou and Zhou, 2021). High oxidative stress has been shown to impair cellular function and angiogenesis (Yin and Xu, 2011; Hu and Wang, 2018). NADPH oxidase 2 (Nox2), as part of the NADPH oxidase complex, also known as Cybb, is a major source of ROS in endothelial cells, a pro-inflammatory factor related to interactions between neutrophils and macrophages, and plays a crucial role in angiogenesis (Hahner and Moll, 2020; Chen and Sun, 2021). Previous studies have shown that Cd44 plays an important role in atherosclerotic lesions characterized by VSMC proliferation, which is mainly involved in angiogenesis, endothelial cell proliferation, and migration (Schultz and Rasmussen, 2005; Zhao and Lee, 2008). In our previous study, we analyzed the dataset uploaded by Dunn et al. using bioinformatic analyses and found that the expression of Cd44 was significantly upregulated after 7 days of carotid artery ligation (Zhang and Gu, 2022). Moreover, it is closely related to ferroptosis and has been studied in ulcerative colitis (Cui and Chen, 2021) and various cancers (Liu and Jiang, 2019; Deng and Zheng, 2021; Kozawa and Sekai, 2021). However, the role of Cd44 in ferroptosis and IH remains unknown, this is the innovation of our research. Furthermore, as a cell surface receptor necessary for cellular iron uptake, transferrin receptor (Tfrc) is an essential component of ferroptotic cell death (Luo and Gao, 2020). In a study by Guo, they identified the role of TRIB2 in mitigating oxidative damage by reducing ubiquitination and the availability of Ub, which is necessary for the subsequent degradation of glutathione peroxidase 4 (GPX4). Thus, they elucidated a novel role for TRIB2 in desensitizing ferroptosis via E3 βTrCP, by which it promotes Tfrc ubiquitination and ultimately reduces labile iron in hepatoma cells (Guo and Chen, 2021).

Hub genes are considered to play key roles in many biological processes. Previous studies have confirmed that TFs and miRNAs participate in the pathological process of IH by regulating various target genes. To gain insight into the mechanism of FRGs in IH, we systematically analyzed the hub gene-miRNA and hub gene-TF networks of five hub FRGs 2 and 14 days after ligation. Previous studies have confirmed that TFs can drive cell differentiation (Fong and Tapscott, 2013), as well as dedifferentiation and transdifferentiation (Takahashi and Yamanaka, 2016). Moreover, TFs also control specific pathways, such as the immune response (Singh and Khan, 2014). We first performed TF network analysis on five hub FRGs and found that only Ptgs2, Cd44, and Il1b could be regulated by some or several TFs. Furthermore, we found that NF-κB1 and SP1 played a significant regulatory role. Previous studies have confirmed that NF-κB1, as an important part of TFs, is involved in the regulation of many biological processes. It is also involved in the formation of neointima after vascular injury, mainly by regulating the expression of inflammation-related genes (Yoshimura and Morishita, 2001; Cartwright and Perkins, 2016). In addition, SP1, as a common TF, has been shown to be involved in IH (Yang and Kim, 2013). Whether NF-κB1 and SP1 are involved and how ferroptosis is regulated in IH still needs further exploration. The five hub FRGs can be regulated by different miRNAs using the miRNA network analysis. It is evident that miRNA-335-3p plays an important role because it regulates the expression of three important genes simultaneously. The available literature indicates that there are few studies on miR-335-3p, especially in the cardiovascular field, which mainly focus on cardiac development (Kay and Soltani, 2019), pulmonary hypertension (Fan and Fan, 2020), and atherosclerosis (Hildebrandt and Kirchner, 2021). Moreover, Sun et al. found that the overexpression of miR-185-5p could suppress the proliferation and migration of VSMCs by targeting FRS2 (Sun and Li, 2021). During VSMC phenotype switching, it was demonstrated that miR-221-3P enhanced VSMC growth *in vitro* and aggravated IH in balloon-injured carotid arteries (Davis and Hilyard, 2009; Liu and Cheng, 2009). Following miR-222-5p knockdown, the proliferative and migratory abilities were inhibited in VSMCs induced by ox-LDL (Liu and Jiang, 2022). To further verify the accuracy of our bioinformatic analysis results, we verified the mRNA and protein levels of the hub FRGs using RT-qPCR and IHC, respectively, and we found that there were only four hub FRGs, including Il1b, Ptgs2, Cybb, and Cd44 that were significantly differentiated in IH induced by carotid artery ligation.

To the best of our knowledge, there are only a few studies on ferroptosis in the context of IH. In the present study, we provided a stepping stone for research regarding this aspect by unveiling the link between ferroptosis and IH. Taken together, our findings provided molecular-level evidence that FRGs at 2 and 14 days after ligation rely on similar and different molecular mechanisms, respectively. Importantly, key ferroptosis-related DEGs were identified during the development of IH; this indicates the existence of common targets and pathways between ferroptosis and IH. However, the limitation of this study was that the regulatory aspect of some signaling pathways was underemphasized, most likely because we only used microarrays, qPCR, and *in silico* tools for analyses. In the present study, we provide some new insights about the underlying mechanism of IH by exploring the key FRGs in the development of IH. Importantly, our results have good novelty and provide key clue to further study the potential target in the next work. The future study will focus on the role of the key FRGs at the cellular and animal levels, to further study the role and mechanism of ferroptosis in the occurrence and development of IH, it is necessary to determine the functions of FRGs and the pathways involved in ferroptosis, as well as the functions and mechanisms underlying the actions of FRGs in this disease.

## 5 Conclusion

To the best of our knowledge, the present study is the first to explore the role of ferroptosis in vascular IH. Our results suggest that these hub FRGs are involved in the occurrence and development of intimal formation. Importantly, our study provides a rich source of targets and pathways that can be further explored to obtain an in-depth picture of the role of ferroptosis in IH.

## Data Availability

The datasets presented in this study can be found in online repositories. The names of the repository/repositories and accession number(s) can be found below: https://www.ncbi.nlm.nih.gov/geo/, GES182291.
