# Supplementary figures and images for: Expression profiles and functions of ferroptosis-related genes in intimal hyperplasia induced by carotid artery ligation in mice
Source: Front Genet. 2022 Aug 30;13:964458. doi: 10.3389/fgene.2022.964458 (PMC9468614; doi:10.3389/fgene.2022.964458)

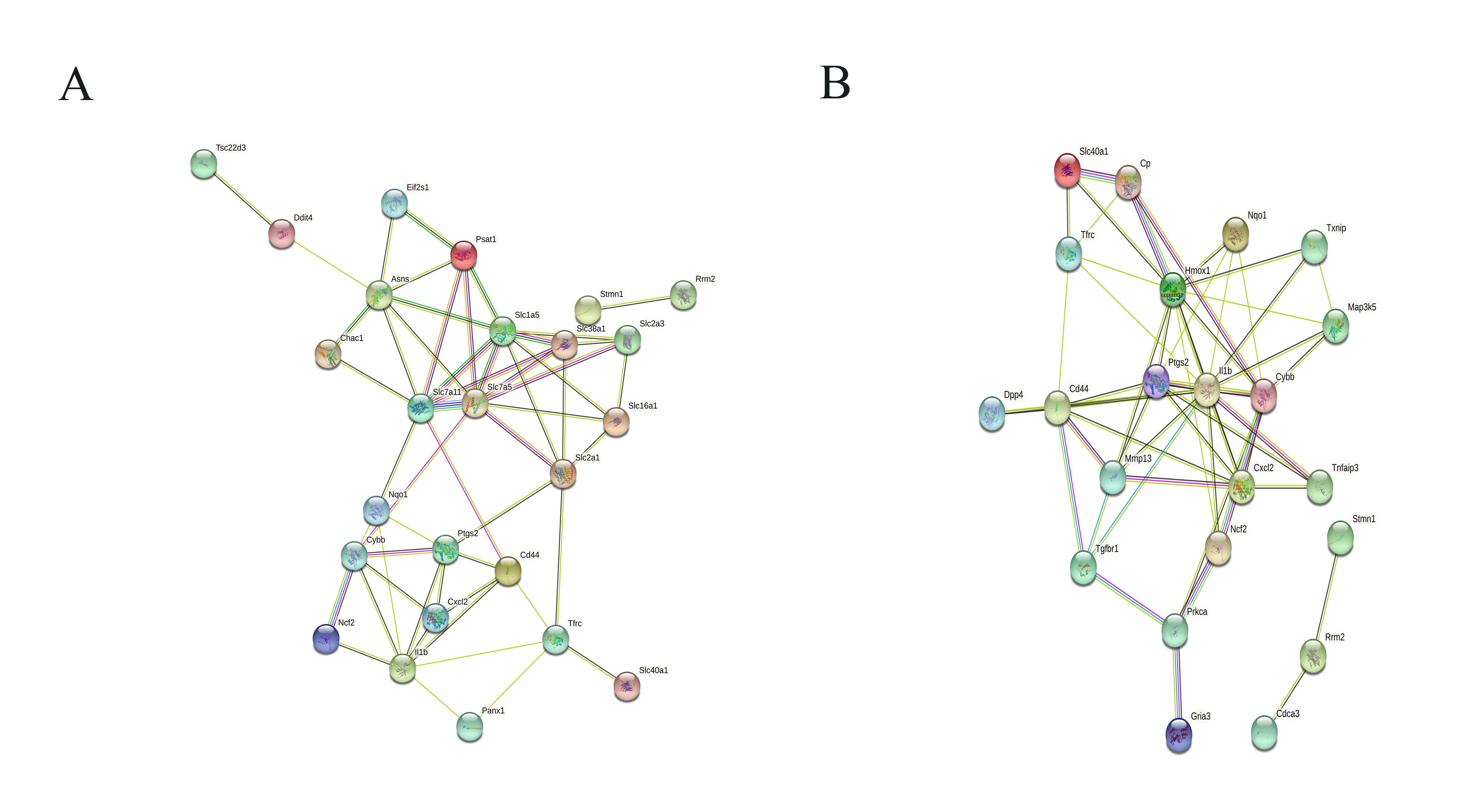

Supplement: Supplementary file 1 [file Image1.jpg]
